# Supplementary material for: MetaRibo-Seq measures translation in microbiomes
Source: Nat Commun. 2020 Jun 29;11:3268. doi: 10.1038/s41467-020-17081-z (PMC7324362; doi:10.1038/s41467-020-17081-z)
Supplement: Supplementary file 10 — Supplementary Data 7 [file 41467_2020_17081_MOESM10_ESM.zip › File2/Confidence_High_Taxonomy/29784_out.krona.html]

Javascript must be enabled to view this page.

members
magnitude
magnitudeUnassigned
count
unassigned
taxon
rank

29784\_out

40

superkingdom
40
2

phylum
40
1239

class
40
186801

186802
order
40

family
40
186803

40
genus
841

40
species

SRS011084\_contig\_number\_34133SRS013158\_contig\_number\_contig-100\_231.42737SRS014287\_contig\_number\_10697SRS014412\_contig\_number\_6718SRS015065\_contig\_number\_32098SRS015190\_contig\_number\_contig-100\_442.98356SRS015431\_contig\_number\_61509SRS015578\_contig\_number\_26868SRS015579\_contig\_number\_14807SRS018817\_contig\_number\_7467SRS019068\_contig\_number\_46994SRS019397\_contig\_number\_3840SRS021484\_contig\_number\_13036SRS024549\_contig\_number\_4543SRS043001\_contig\_number\_7877SRS043411\_contig\_number\_10865SRS045739\_contig\_number\_4389SRS054956\_contig\_number\_contig-100\_498.93932SRS055017\_contig\_number\_31640SRS058070\_contig\_number\_26186SRS063040\_contig\_number\_contig-100\_250.265240SRS065504\_contig\_number\_28624SRS075341\_contig\_number\_18619SRS075716\_contig\_number\_966SRS076756\_contig\_number\_contig-100\_896.123403SRS076929\_contig\_number\_22432SRS077641\_contig\_number\_9567SRS098061\_contig\_number\_9887SRS098514\_contig\_number\_contig-100\_1083.1083SRS098881\_contig\_number\_6459SRS104485\_contig\_number\_4080SRS105153\_contig\_number\_39372SRS142781\_contig\_number\_11274SRS143070\_contig\_number\_11237SRS143417\_contig\_number\_19136SRS147919\_contig\_number\_contig-100\_544.117411SRS148091\_contig\_number\_10274SRS148784\_contig\_number\_contig-100\_179.108491SRS149181\_contig\_number\_30895SRS149244\_contig\_number\_1046
166486
